# Asphyxia Neonatorum

**Published:** 1888-03

**Authors:** 


### ASPHYXIA NEONATORUM.

Aconite.—Child is hot, purple-hued, pulseless and breathless, or nearly so.

Antimonium-tart.—Much rattling of mucus; pale, with gasping, pulseless.

Belladonna.—Face very red and the eyeballs are greatly injected.

Laurocerasus.—Blueness of face, twitching of muscles of face and gasping with rattling breathing.

Opium.—Pale and breathless, cord still pulsates.

